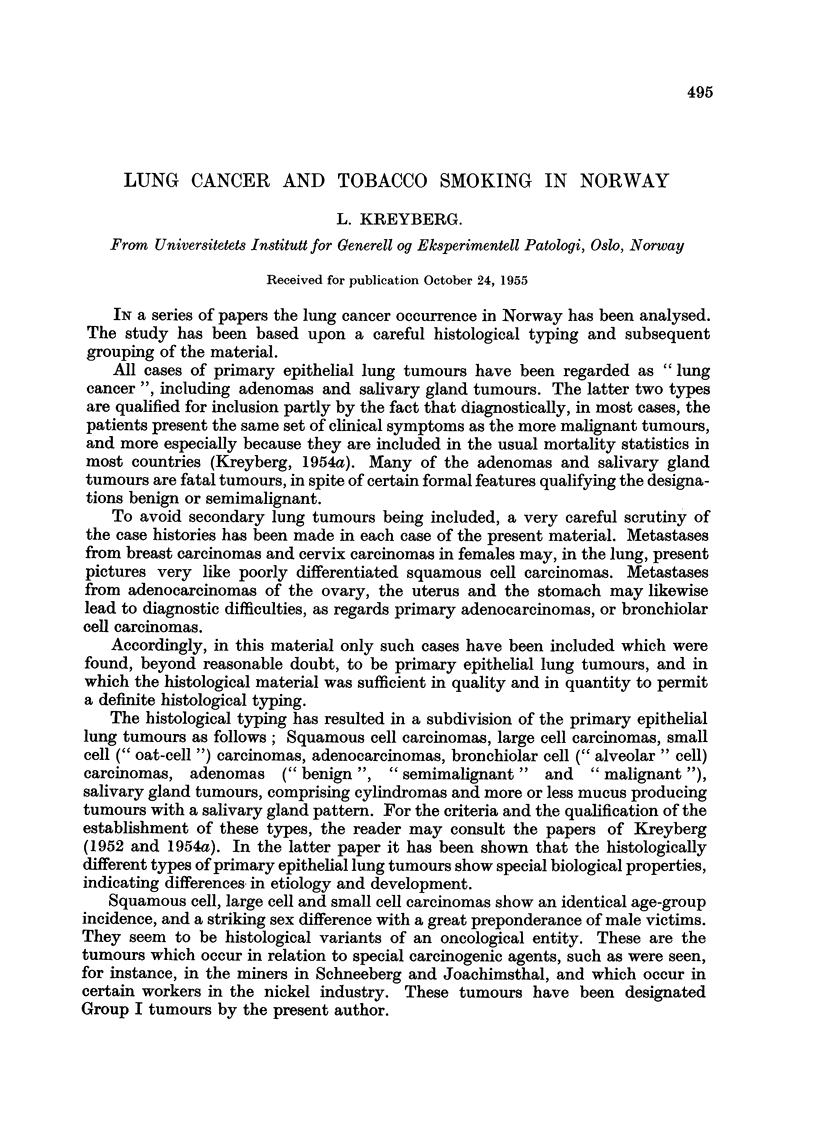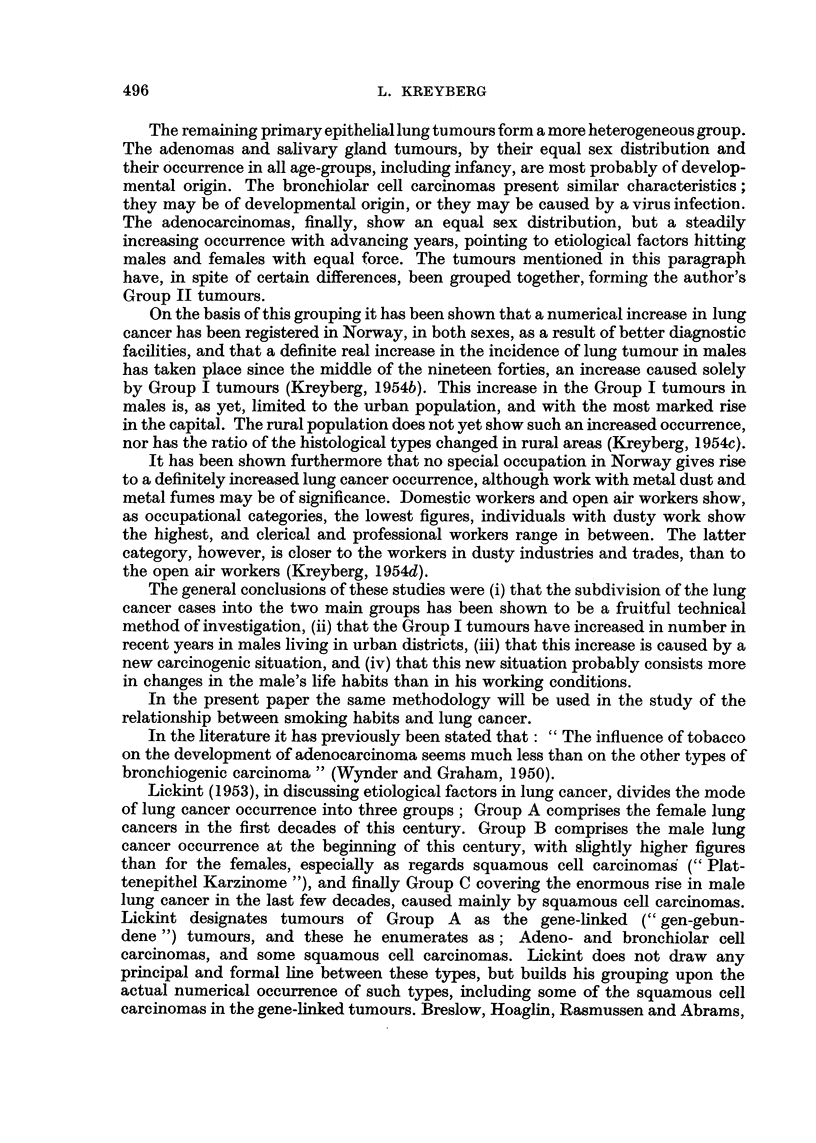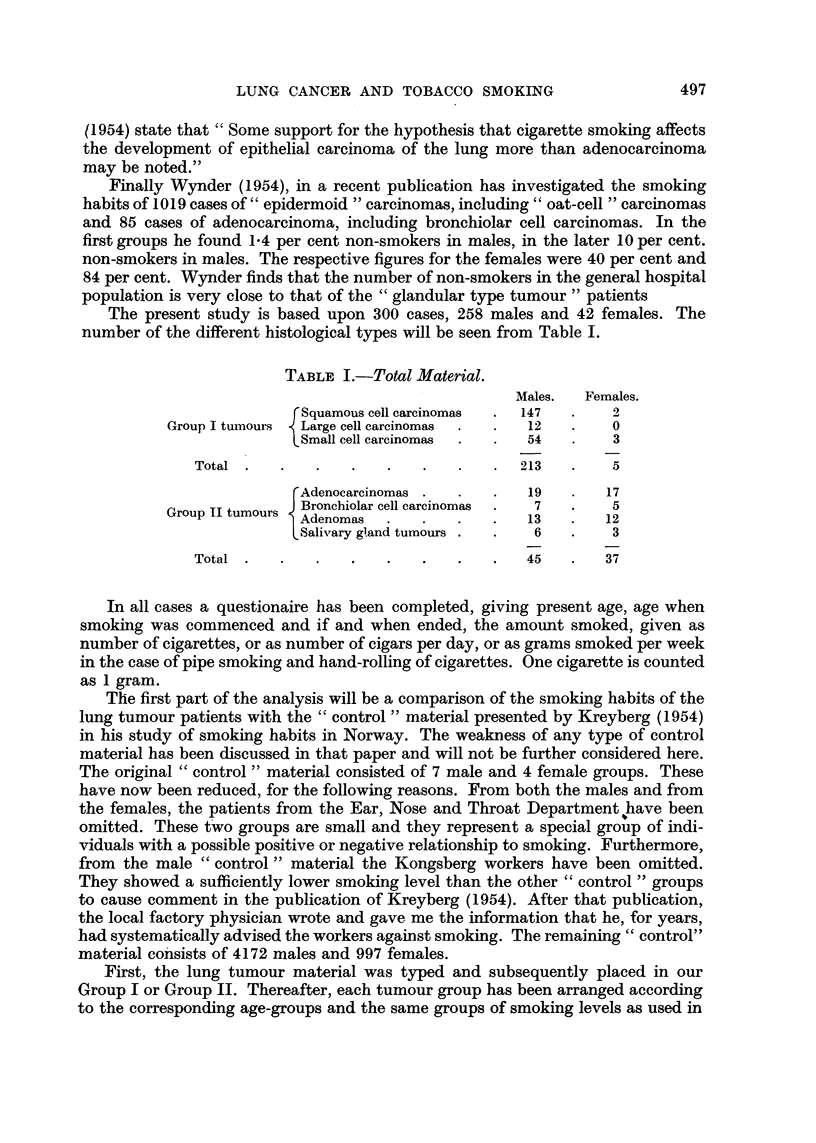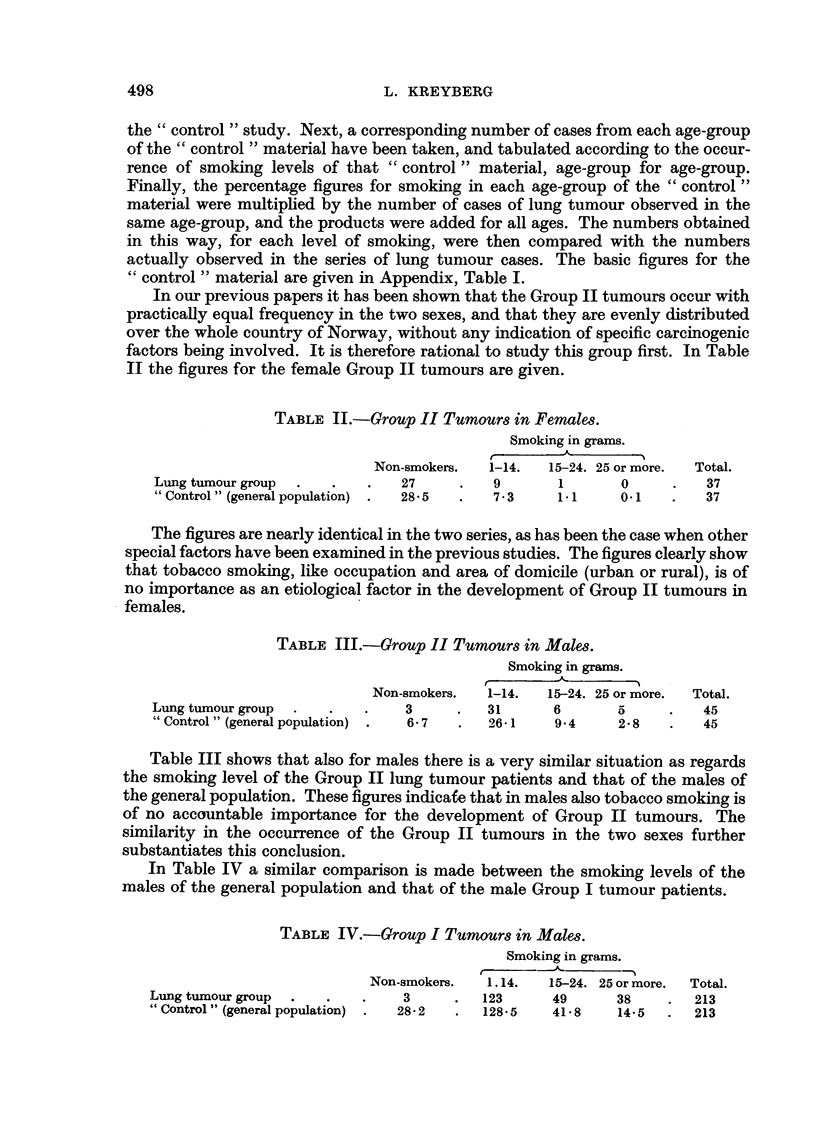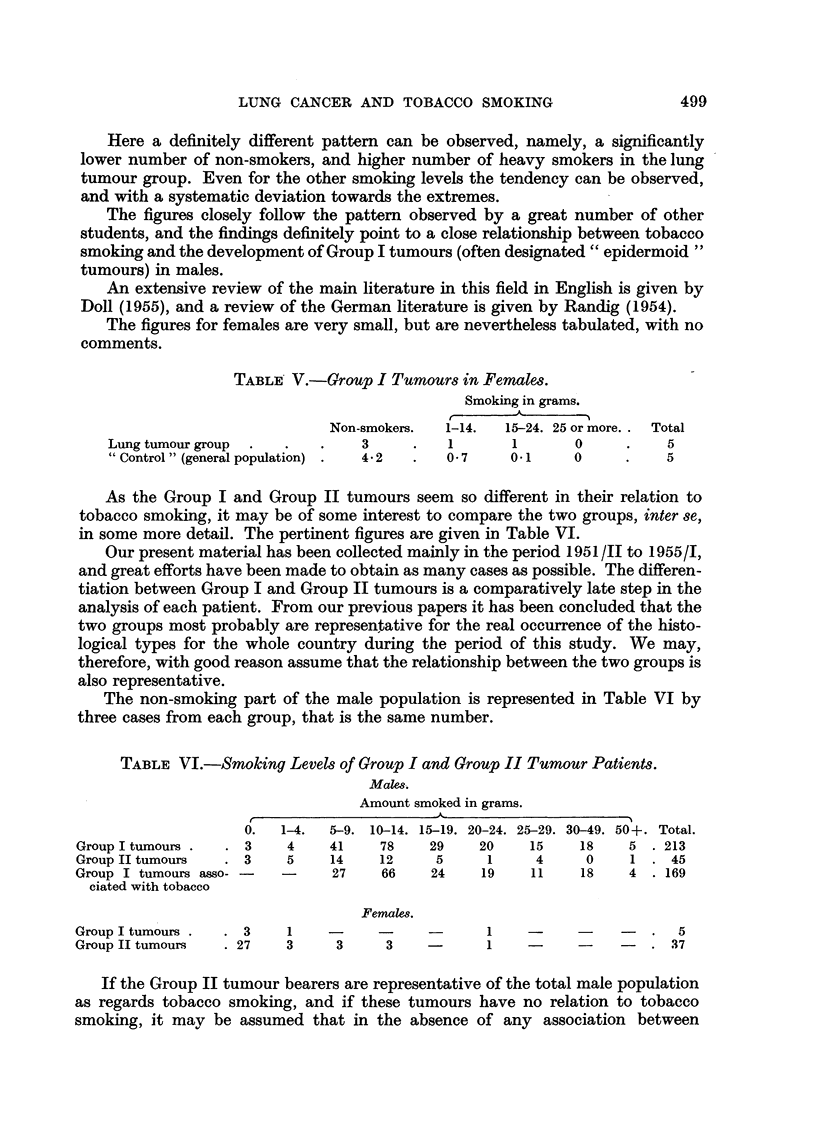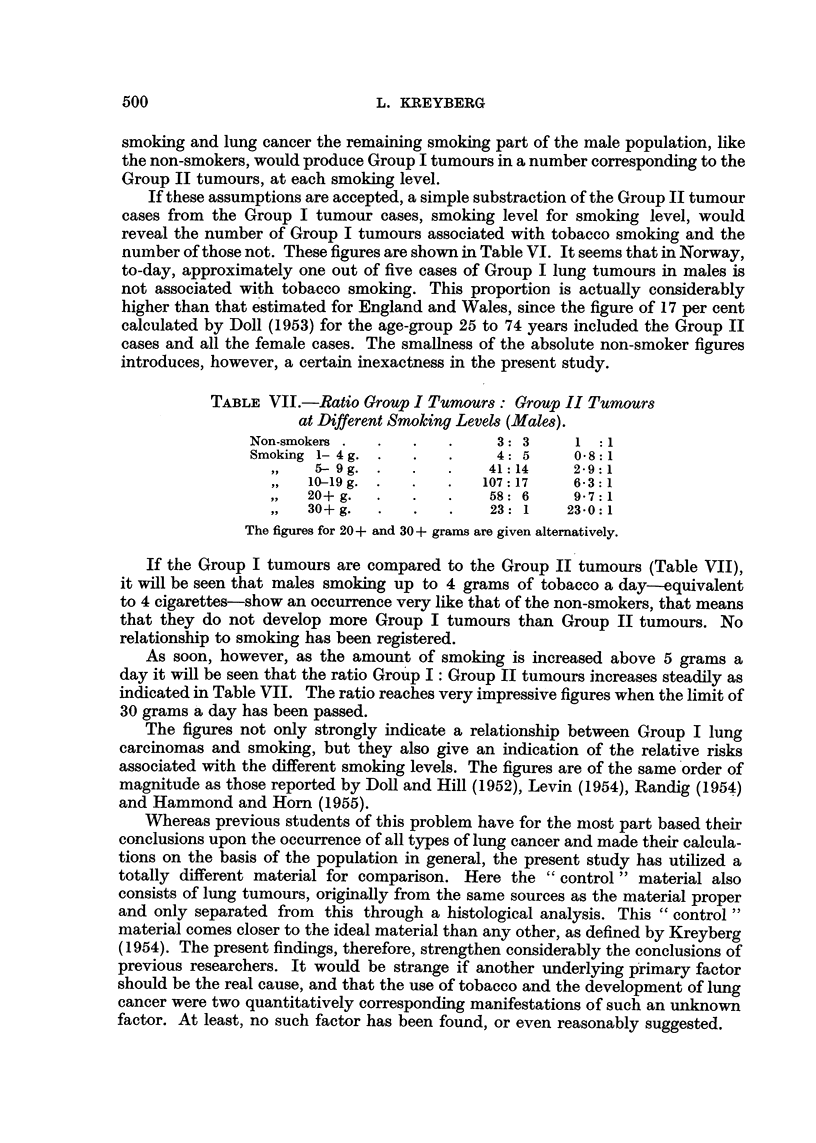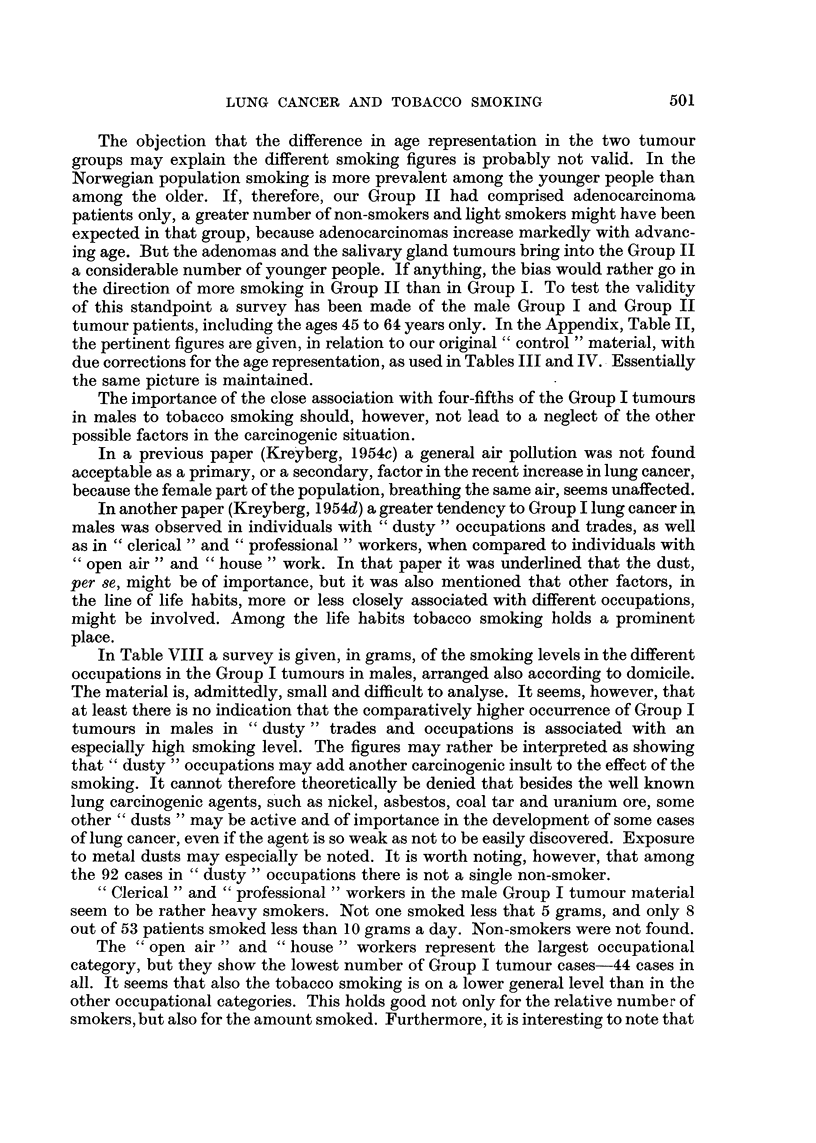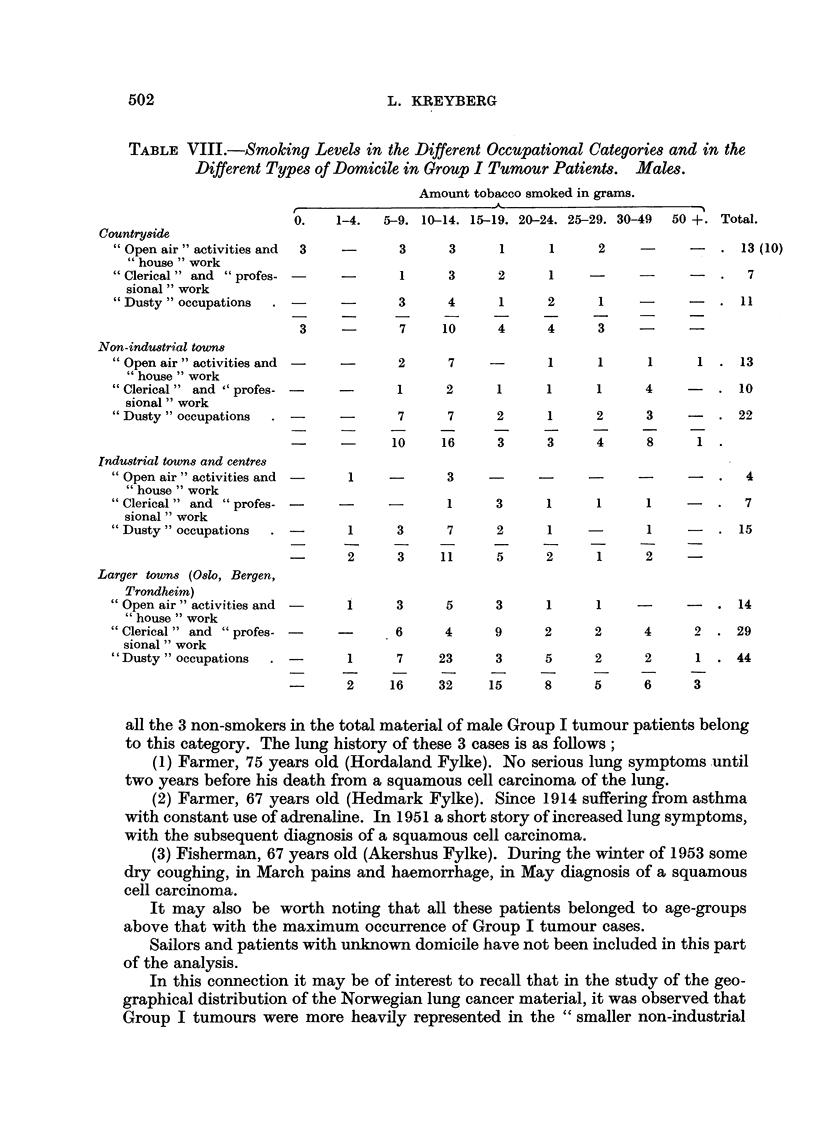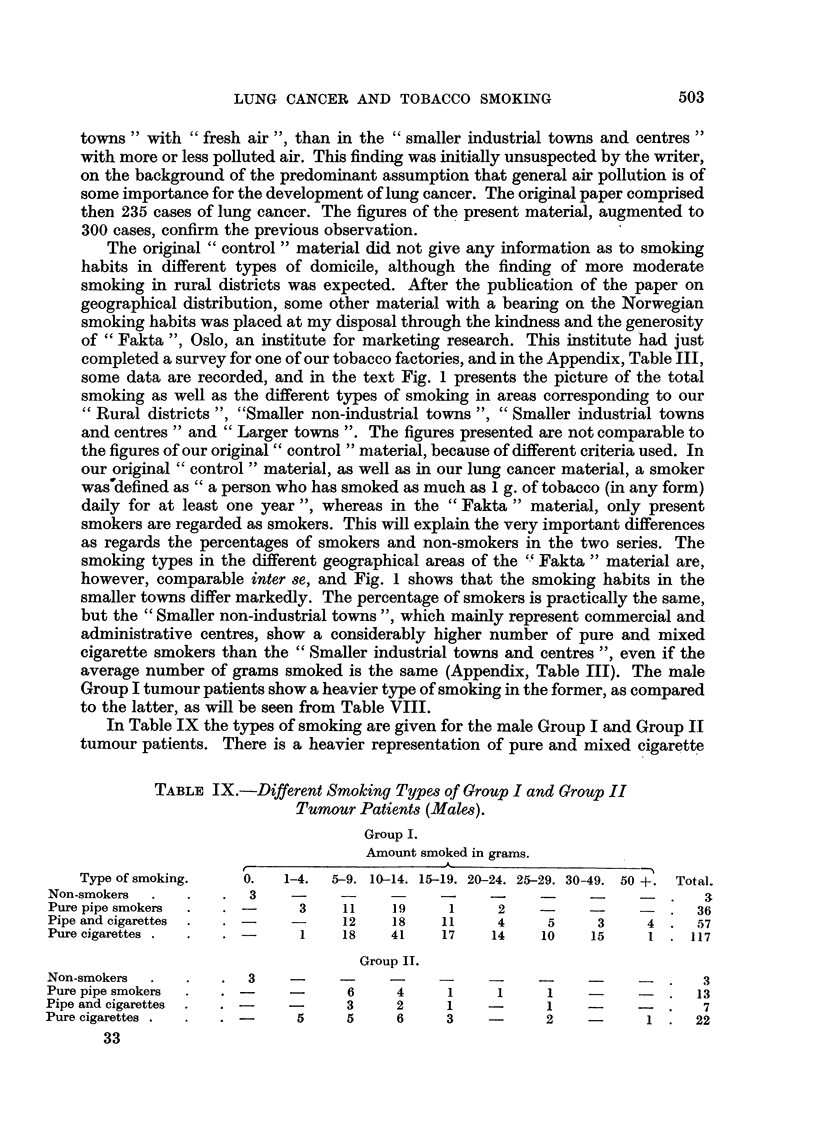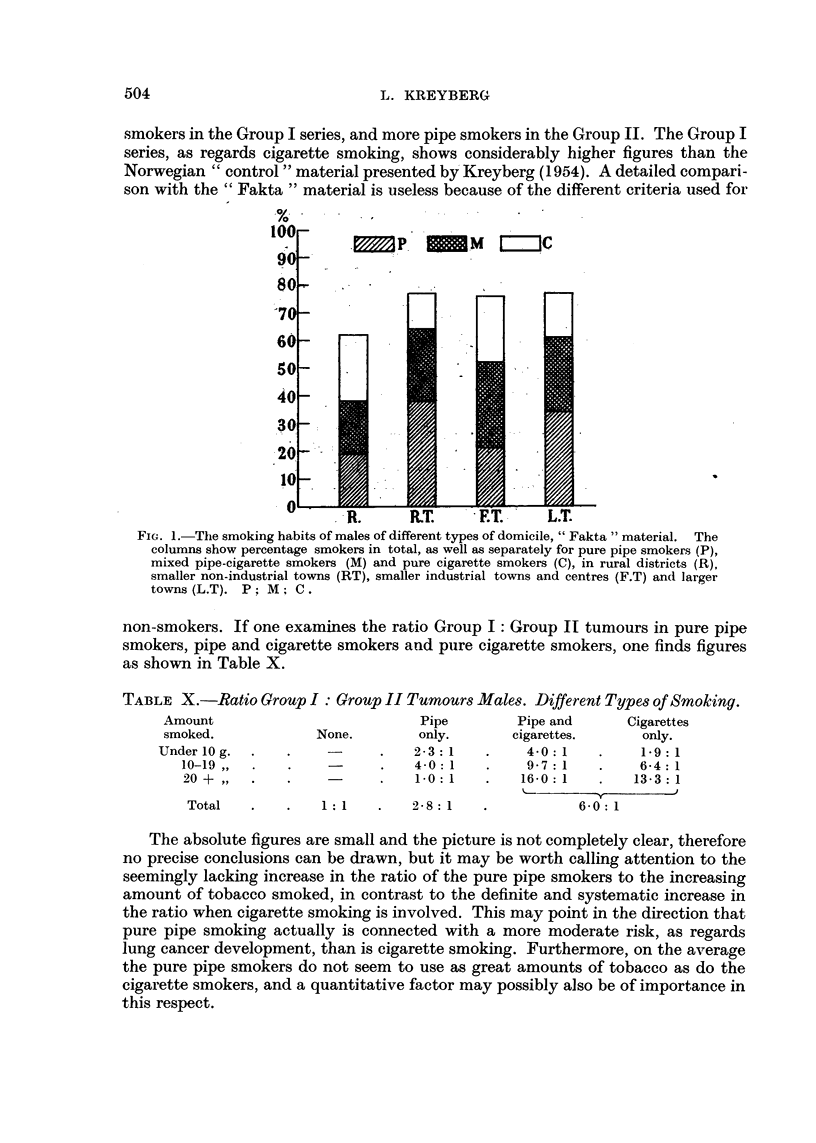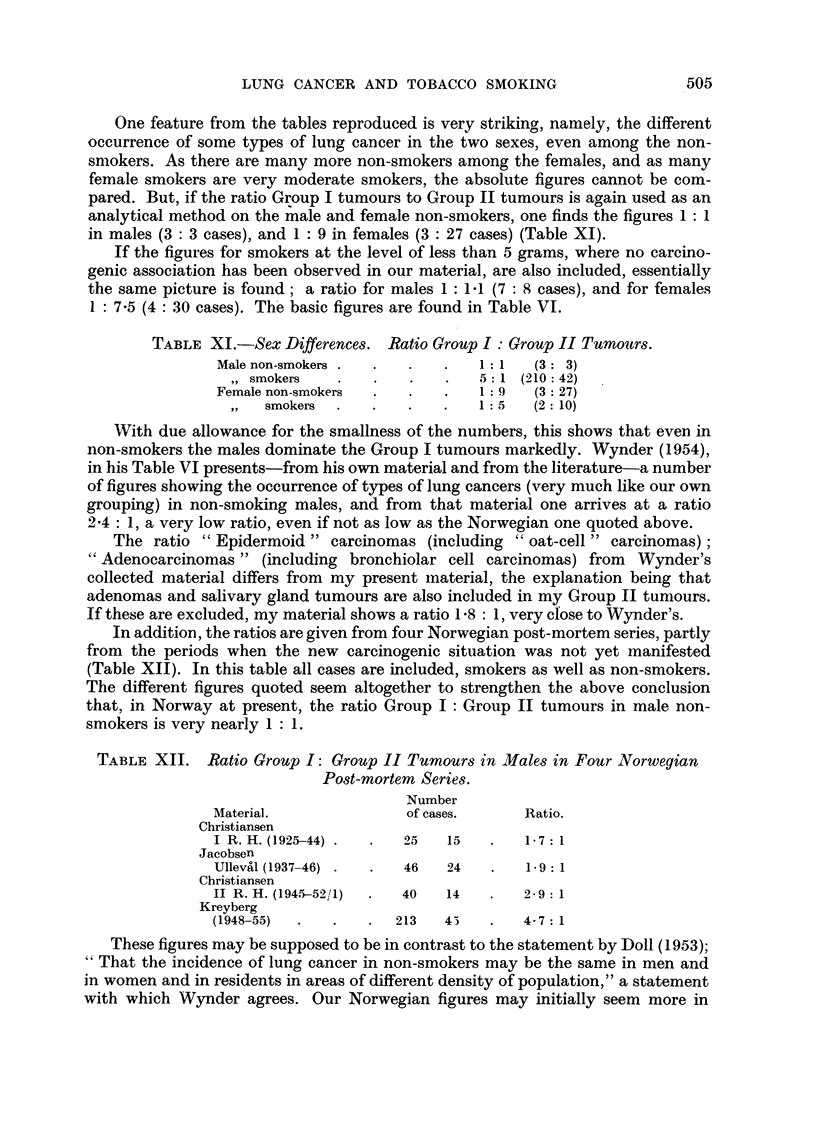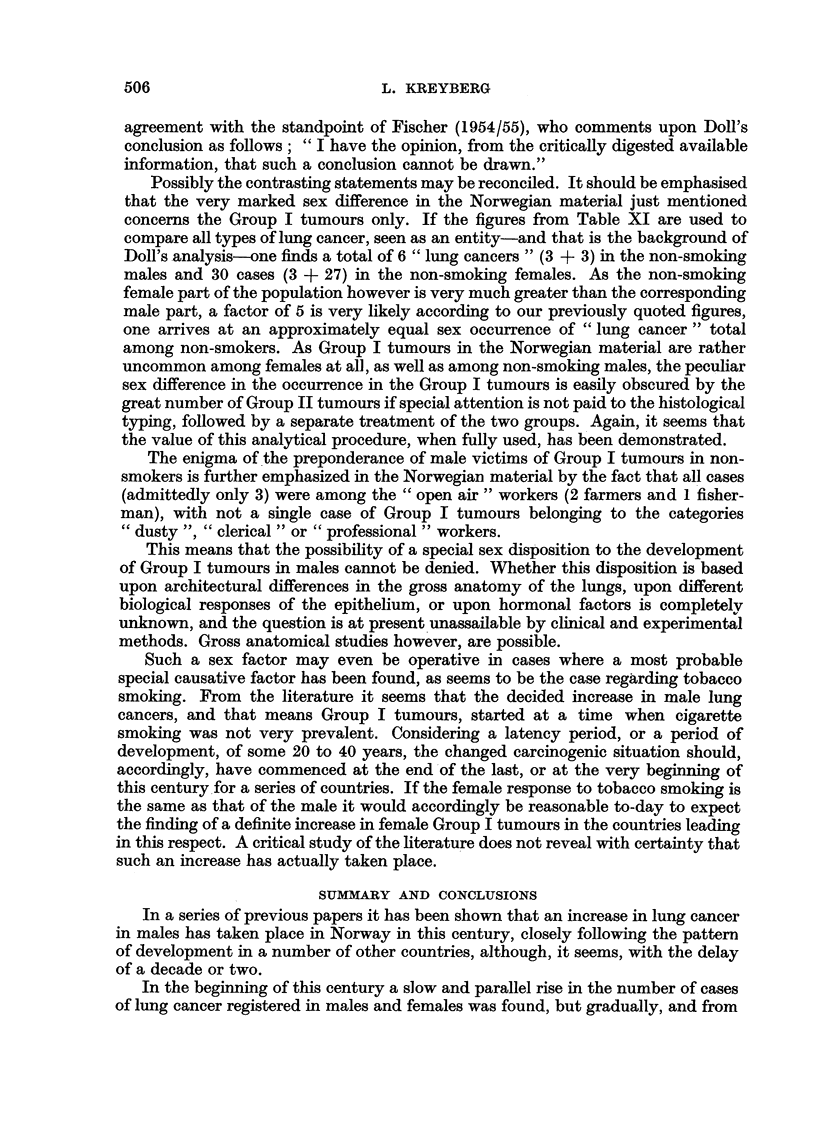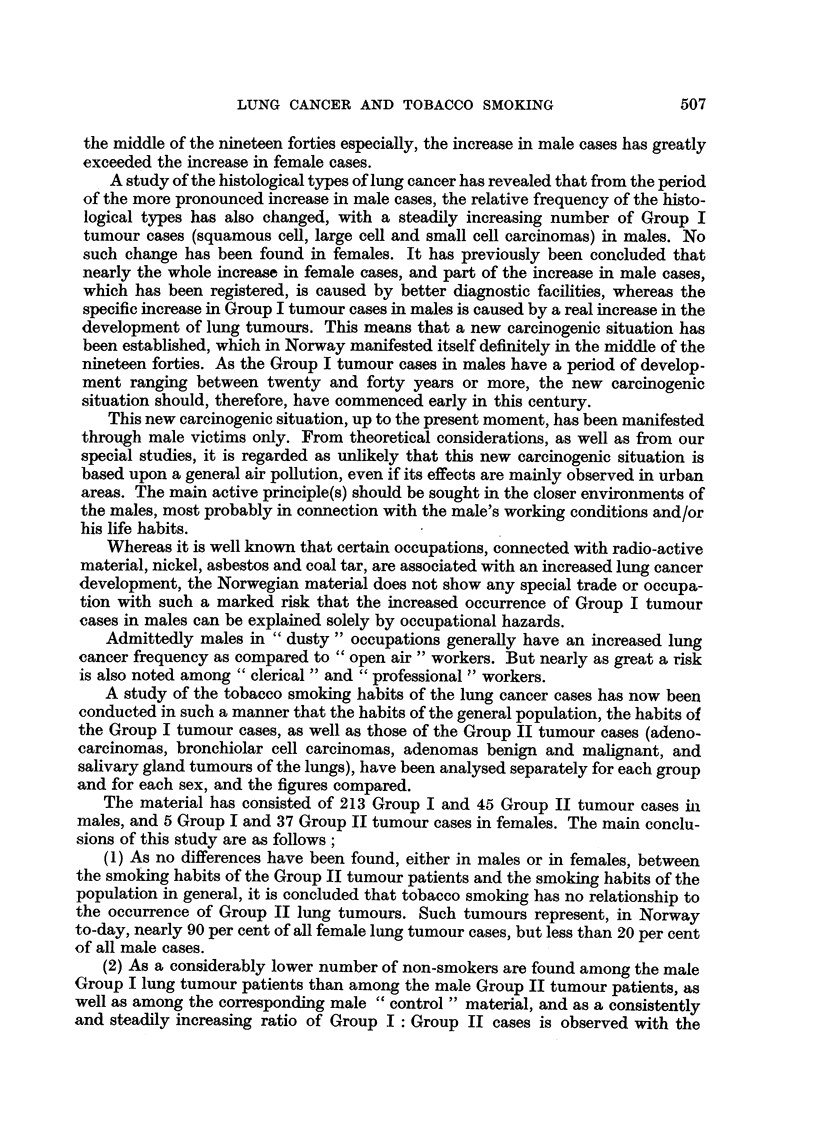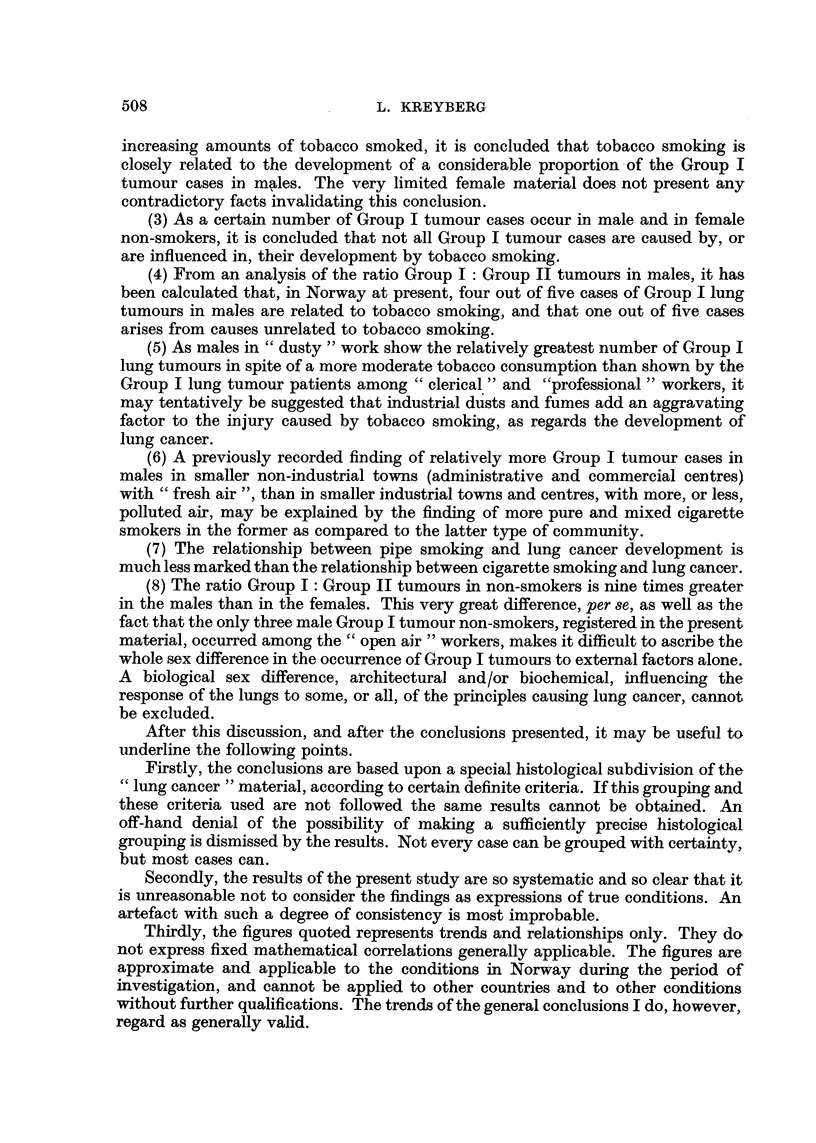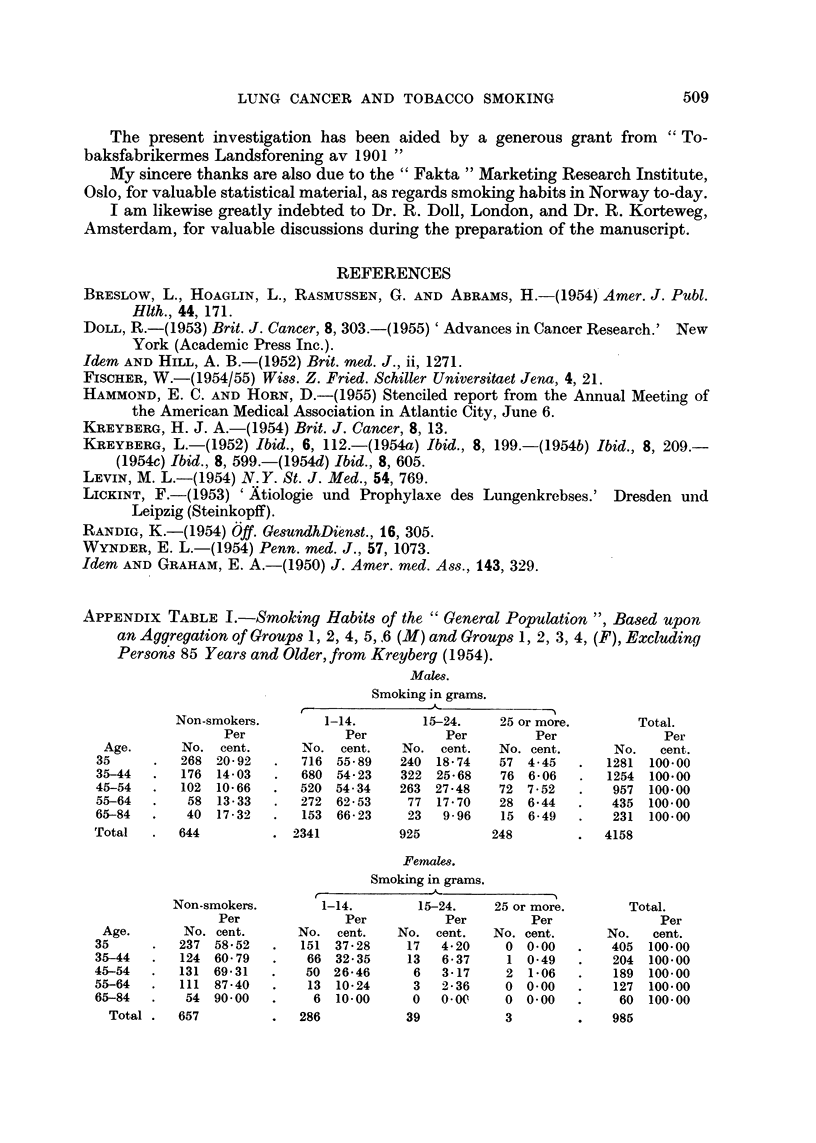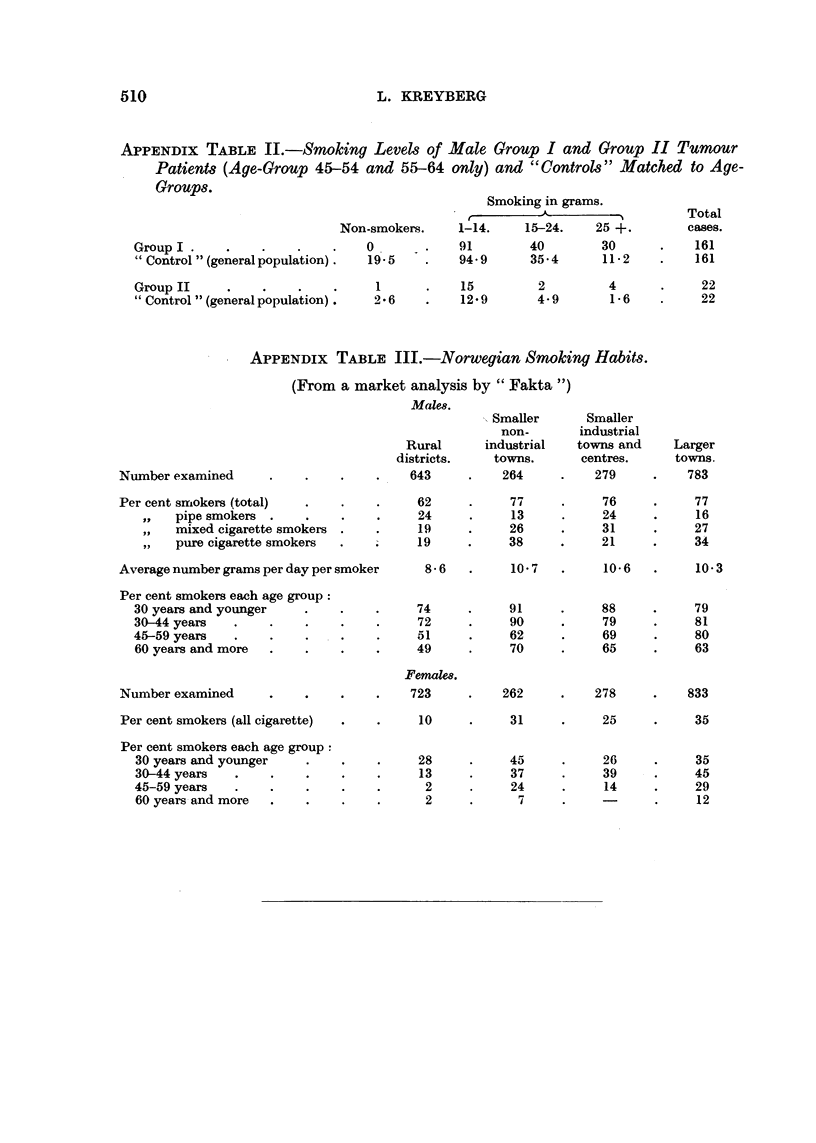# Lung Cancer and Tobacco Smoking in Norway

**DOI:** 10.1038/bjc.1955.52

**Published:** 1955-12

**Authors:** L. Kreyberg


### LUNG CANCER AND TOBACCO SMOKING IN NORWAY

L. KREYBERG.

*From Universitetets Institutt for Generell og Eksperimentell Patologi, Oslo, Norway*

Received for publication October 24, 1955

IN a series of papers the lung cancer occurrence in Norway has been analysed. The study has been based upon a careful histological typing and subsequent grouping of the material.

All cases of primary epithelial lung tumours have been regarded as " lung cancer ", including adenomas and salivary gland tumours. The latter two types are qualified for inclusion partly by the fact that diagnostically, in most cases, the patients present the same set of clinical symptoms as the more malignant tumours, and more especially because they are included in the usual mortality statistics in most countries (Kreyberg, 1954*a*). Many of the adenomas and salivary gland tumours are fatal tumours, in spite of certain formal features qualifying the designations benign or semimalignant.

To avoid secondary lung tumours being included, a very careful scrutiny of the case histories has been made in each case of the present material. Metastases from breast carcinomas and cervix carcinomas in females may, in the lung, present pictures very like poorly differentiated squamous cell carcinomas. Metastases from adenocarcinomas of the ovary, the uterus and the stomach may likewise lead to diagnostic difficulties, as regards primary adenocarcinomas, or bronchiolar cell carcinomas.

Accordingly, in this material only such cases have been included which were found, beyond reasonable doubt, to be primary epithelial lung tumours, and in which the histological material was sufficient in quality and in quantity to permit a definite histological typing.

The histological typing has resulted in a subdivision of the primary epithelial lung tumours as follows ; Squamous cell carcinomas, large cell carcinomas, small cell (" oat-cell ") carcinomas, adenocarcinomas, bronchiolar cell (" alveolar " cell) carcinomas, adenomas (" benign ", " semimalignant " and " malignant "), salivary gland tumours, comprising cylindromas and more or less mucus producing tumours with a salivary gland pattern. For the criteria and the qualification of the establishment of these types, the reader may consult the papers of Kreyberg (1952 and 1954*a*). In the latter paper it has been shown that the histologically different types of primary epithelial lung tumours show special biological properties, indicating differences in etiology and development.

Squamous cell, large cell and small cell carcinomas show an identical age-group incidence, and a striking sex difference with a great preponderance of male victims. They seem to be histological variants of an oncological entity. These are the tumours which occur in relation to special carcinogenic agents, such as were seen, for instance, in the miners in Schneeberg and Joachimsthal, and which occur in certain workers in the nickel industry. These tumours have been designated Group I tumours by the present author.

The remaining primary epithelial lung tumours form a more heterogeneous group. The adenomas and salivary gland tumours, by their equal sex distribution and their occurrence in all age-groups, including infancy, are most probably of developmental origin. The bronchiolar cell carcinomas present similar characteristics; they may be of developmental origin, or they may be caused by a virus infection. The adenocarcinomas, finally, show an equal sex distribution, but a steadily increasing occurrence with advancing years, pointing to etiological factors hitting males and females with equal force. The tumours mentioned in this paragraph have, in spite of certain differences, been grouped together, forming the author's Group II tumours.

On the basis of this grouping it has been shown that a numerical increase in lung cancer has been registered in Norway, in both sexes, as a result of better diagnostic facilities, and that a definite real increase in the incidence of lung tumour in males has taken place since the middle of the nineteen forties, an increase caused solely by Group I tumours (Kreyberg, 1954*b*). This increase in the Group I tumours in males is, as yet, limited to the urban population, and with the most marked rise in the capital. The rural population does not yet show such an increased occurrence, nor has the ratio of the histological types changed in rural areas (Kreyberg, 1954*c*).

It has been shown furthermore that no special occupation in Norway gives rise to a definitely increased lung cancer occurrence, although work with metal dust and metal fumes may be of significance. Domestic workers and open air workers show, as occupational categories, the lowest figures, individuals with dusty work show the highest, and clerical and professional workers range in between. The latter category, however, is closer to the workers in dusty industries and trades, than to the open air workers (Kreyberg, 1954*d*).

The general conclusions of these studies were (i) that the subdivision of the lung cancer cases into the two main groups has been shown to be a fruitful technical method of investigation, (ii) that the Group I tumours have increased in number in recent years in males living in urban districts, (iii) that this increase is caused by a new carcinogenic situation, and (iv) that this new situation probably consists more in changes in the male's life habits than in his working conditions.

In the present paper the same methodology will be used in the study of the relationship between smoking habits and lung cancer.

In the literature it has previously been stated that: " The influence of tobacco on the development of adenocarcinoma seems much less than on the other types of bronchiogenic carcinoma " (Wynder and Graham, 1950).

Lickint (1953), in discussing etiological factors in lung cancer, divides the mode of lung cancer occurrence into three groups; Group A comprises the female lung cancers in the first decades of this century. Group B comprises the male lung cancer occurrence at the beginning of this century, with slightly higher figures than for the females, especially as regards squamous cell carcinomas (" Plattenepithel Karzinome "), and finally Group C covering the enormous rise in male lung cancer in the last few decades, caused mainly by squamous cell carcinomas. Lickint designates tumours of Group A as the gene-linked (" gen-gebundene ") tumours, and these he enumerates as; Adeno- and bronchiolar cell carcinomas, and some squamous cell carcinomas. Lickint does not draw any principal and formal line between these types, but builds his grouping upon the actual numerical occurrence of such types, including some of the squamous cell carcinomas in the gene-linked tumours. Breslow, Hoaglin, Rasmussen and Abrams,

(1954) state that " Some support for the hypothesis that cigarette smoking affects the development of epithelial carcinoma of the lung more than adenocarcinoma may be noted."

Finally Wynder (1954), in a recent publication has investigated the smoking habits of 1019 cases of " epidermoid " carcinomas, including " oat-cell " carcinomas and 85 cases of adenocarcinoma, including bronchiolar cell carcinomas. In the first groups he found 1·4 per cent non-smokers in males, in the later 10 per cent. non-smokers in males. The respective figures for the females were 40 per cent and 84 per cent. Wynder finds that the number of non-smokers in the general hospital population is very close to that of the " glandular type tumour " patients

The present study is based upon 300 cases, 258 males and 42 females. The number of the different histological types will be seen from Table I.

TABLE I.—*Total Material.*

| | | Males. | Females. |
|---|---|---|---|
| Group I tumours | Squamous cell carcinomas | 147 | 2 |
| | Large cell carcinomas | 12 | 0 |
| | Small cell carcinomas | 54 | 3 |
| Total | | 213 | 5 |
| Group II tumours | Adenocarcinomas | 19 | 17 |
| | Bronchiolar cell carcinomas | 7 | 5 |
| | Adenomas | 13 | 12 |
| | Salivary gland tumours | 6 | 3 |
| Total | | 45 | 37 |

In all cases a questionaire has been completed, giving present age, age when smoking was commenced and if and when ended, the amount smoked, given as number of cigarettes, or as number of cigars per day, or as grams smoked per week in the case of pipe smoking and hand-rolling of cigarettes. One cigarette is counted as 1 gram.

The first part of the analysis will be a comparison of the smoking habits of the lung tumour patients with the " control " material presented by Kreyberg (1954) in his study of smoking habits in Norway. The weakness of any type of control material has been discussed in that paper and will not be further considered here. The original " control " material consisted of 7 male and 4 female groups. These have now been reduced, for the following reasons. From both the males and from the females, the patients from the Ear, Nose and Throat Department have been omitted. These two groups are small and they represent a special group of individuals with a possible positive or negative relationship to smoking. Furthermore, from the male " control " material the Kongsberg workers have been omitted. They showed a sufficiently lower smoking level than the other " control " groups to cause comment in the publication of Kreyberg (1954). After that publication, the local factory physician wrote and gave me the information that he, for years, had systematically advised the workers against smoking. The remaining " control" material consists of 4172 males and 997 females.

First, the lung tumour material was typed and subsequently placed in our Group I or Group II. Thereafter, each tumour group has been arranged according to the corresponding age-groups and the same groups of smoking levels as used in the " control " study. Next, a corresponding number of cases from each age-group of the " control " material have been taken, and tabulated according to the occurrence of smoking levels of that " control " material, age-group for age-group. Finally, the percentage figures for smoking in each age-group of the " control " material were multiplied by the number of cases of lung tumour observed in the same age-group, and the products were added for all ages. The numbers obtained in this way, for each level of smoking, were then compared with the numbers actually observed in the series of lung tumour cases. The basic figures for the " control " material are given in Appendix, Table I.

In our previous papers it has been shown that the Group II tumours occur with practically equal frequency in the two sexes, and that they are evenly distributed over the whole country of Norway, without any indication of specific carcinogenic factors being involved. It is therefore rational to study this group first. In Table II the figures for the female Group II tumours are given.

TABLE II.—*Group II Tumours in Females.*

| | Non-smokers. | Smoking in grams. 1–14. | 15–24. | 25 or more. | Total. |
|---|---|---|---|---|---|
| Lung tumour group | 27 | 9 | 1 | 0 | 37 |
| " Control " (general population) | 28·5 | 7·3 | 1·1 | 0·1 | 37 |

The figures are nearly identical in the two series, as has been the case when other special factors have been examined in the previous studies. The figures clearly show that tobacco smoking, like occupation and area of domicile (urban or rural), is of no importance as an etiological factor in the development of Group II tumours in females.

TABLE III.—*Group II Tumours in Males.*

| | Non-smokers. | Smoking in grams. 1–14. | 15–24. | 25 or more. | Total. |
|---|---|---|---|---|---|
| Lung tumour group | 3 | 31 | 6 | 5 | 45 |
| " Control " (general population) | 6·7 | 26·1 | 9·4 | 2·8 | 45 |

Table III shows that also for males there is a very similar situation as regards the smoking level of the Group II lung tumour patients and that of the males of the general population. These figures indicate that in males also tobacco smoking is of no accountable importance for the development of Group II tumours. The similarity in the occurrence of the Group II tumours in the two sexes further substantiates this conclusion.

In Table IV a similar comparison is made between the smoking levels of the males of the general population and that of the male Group I tumour patients.

TABLE IV.—*Group I Tumours in Males.*

| | Non-smokers. | Smoking in grams. 1.14. | 15–24. | 25 or more. | Total. |
|---|---|---|---|---|---|
| Lung tumour group | 3 | 123 | 49 | 38 | 213 |
| " Control " (general population) | 28·2 | 128·5 | 41·8 | 14·5 | 213 |

Here a definitely different pattern can be observed, namely, a significantly lower number of non-smokers, and higher number of heavy smokers in the lung tumour group. Even for the other smoking levels the tendency can be observed, and with a systematic deviation towards the extremes.

The figures closely follow the pattern observed by a great number of other students, and the findings definitely point to a close relationship between tobacco smoking and the development of Group I tumours (often designated "epidermoid" tumours) in males.

An extensive review of the main literature in this field in English is given by Doll (1955), and a review of the German literature is given by Randig (1954).

The figures for females are very small, but are nevertheless tabulated, with no comments.

TABLE V.—*Group I Tumours in Females.*

| | Non-smokers. | Smoking in grams. 1–14. | 15–24. | 25 or more. | Total |
|---|---|---|---|---|---|
| Lung tumour group | 3 | 1 | 1 | 0 | 5 |
| "Control" (general population) | 4·2 | 0·7 | 0·1 | 0 | 5 |

As the Group I and Group II tumours seem so different in their relation to tobacco smoking, it may be of some interest to compare the two groups, *inter se*, in some more detail. The pertinent figures are given in Table VI.

Our present material has been collected mainly in the period 1951/II to 1955/I, and great efforts have been made to obtain as many cases as possible. The differentiation between Group I and Group II tumours is a comparatively late step in the analysis of each patient. From our previous papers it has been concluded that the two groups most probably are representative for the real occurrence of the histological types for the whole country during the period of this study. We may, therefore, with good reason assume that the relationship between the two groups is also representative.

The non-smoking part of the male population is represented in Table VI by three cases from each group, that is the same number.

TABLE VI.—*Smoking Levels of Group I and Group II Tumour Patients.*

| | Amount smoked in grams. 0. | 1–4. | 5–9. | 10–14. | 15–19. | 20–24. | 25–29. | 30–49. | 50+. | Total. |
|---|---|---|---|---|---|---|---|---|---|---|
| *Males.* | | | | | | | | | | |
| Group I tumours | 3 | 4 | 41 | 78 | 29 | 20 | 15 | 18 | 5 | 213 |
| Group II tumours | 3 | 5 | 14 | 12 | 5 | 1 | 4 | 0 | 1 | 45 |
| Group I tumours associated with tobacco | — | — | 27 | 66 | 24 | 19 | 11 | 18 | 4 | 169 |
| *Females.* | | | | | | | | | | |
| Group I tumours | 3 | 1 | — | — | — | 1 | — | — | — | 5 |
| Group II tumours | 27 | 3 | 3 | 3 | — | 1 | — | — | — | 37 |

If the Group II tumour bearers are representative of the total male population as regards tobacco smoking, and if these tumours have no relation to tobacco smoking, it may be assumed that in the absence of any association between smoking and lung cancer the remaining smoking part of the male population, like the non-smokers, would produce Group I tumours in a number corresponding to the Group II tumours, at each smoking level.

If these assumptions are accepted, a simple substraction of the Group II tumour cases from the Group I tumour cases, smoking level for smoking level, would reveal the number of Group I tumours associated with tobacco smoking and the number of those not. These figures are shown in Table VI. It seems that in Norway, to-day, approximately one out of five cases of Group I lung tumours in males is not associated with tobacco smoking. This proportion is actually considerably higher than that estimated for England and Wales, since the figure of 17 per cent calculated by Doll (1953) for the age-group 25 to 74 years included the Group II cases and all the female cases. The smallness of the absolute non-smoker figures introduces, however, a certain inexactness in the present study.

TABLE VII.—*Ratio Group I Tumours : Group II Tumours at Different Smoking Levels (Males).*

| | | |
|---|---|---|
| Non-smokers . . . . | 3 : 3 | 1 : 1 |
| Smoking 1– 4 g. . . . | 4 : 5 | 0·8 : 1 |
| „ 5– 9 g. . . . | 41 : 14 | 2·9 : 1 |
| „ 10–19 g. . . . | 107 : 17 | 6·3 : 1 |
| „ 20+ g. . . . | 58 : 6 | 9·7 : 1 |
| „ 30+ g. . . . | 23 : 1 | 23·0 : 1 |

The figures for 20+ and 30+ grams are given alternatively.

If the Group I tumours are compared to the Group II tumours (Table VII), it will be seen that males smoking up to 4 grams of tobacco a day—equivalent to 4 cigarettes—show an occurrence very like that of the non-smokers, that means that they do not develop more Group I tumours than Group II tumours. No relationship to smoking has been registered.

As soon, however, as the amount of smoking is increased above 5 grams a day it will be seen that the ratio Group I : Group II tumours increases steadily as indicated in Table VII. The ratio reaches very impressive figures when the limit of 30 grams a day has been passed.

The figures not only strongly indicate a relationship between Group I lung carcinomas and smoking, but they also give an indication of the relative risks associated with the different smoking levels. The figures are of the same order of magnitude as those reported by Doll and Hill (1952), Levin (1954), Randig (1954) and Hammond and Horn (1955).

Whereas previous students of this problem have for the most part based their conclusions upon the occurrence of all types of lung cancer and made their calculations on the basis of the population in general, the present study has utilized a totally different material for comparison. Here the " control " material also consists of lung tumours, originally from the same sources as the material proper and only separated from this through a histological analysis. This " control " material comes closer to the ideal material than any other, as defined by Kreyberg (1954). The present findings, therefore, strengthen considerably the conclusions of previous researchers. It would be strange if another underlying primary factor should be the real cause, and that the use of tobacco and the development of lung cancer were two quantitatively corresponding manifestations of such an unknown factor. At least, no such factor has been found, or even reasonably suggested.

The objection that the difference in age representation in the two tumour groups may explain the different smoking figures is probably not valid. In the Norwegian population smoking is more prevalent among the younger people than among the older. If, therefore, our Group II had comprised adenocarcinoma patients only, a greater number of non-smokers and light smokers might have been expected in that group, because adenocarcinomas increase markedly with advancing age. But the adenomas and the salivary gland tumours bring into the Group II a considerable number of younger people. If anything, the bias would rather go in the direction of more smoking in Group II than in Group I. To test the validity of this standpoint a survey has been made of the male Group I and Group II tumour patients, including the ages 45 to 64 years only. In the Appendix, Table II, the pertinent figures are given, in relation to our original " control " material, with due corrections for the age representation, as used in Tables III and IV. Essentially the same picture is maintained.

The importance of the close association with four-fifths of the Group I tumours in males to tobacco smoking should, however, not lead to a neglect of the other possible factors in the carcinogenic situation.

In a previous paper (Kreÿberg, 1954*c*) a general air pollution was not found acceptable as a primary, or a secondary, factor in the recent increase in lung cancer, because the female part of the population, breathing the same air, seems unaffected.

In another paper (Kreyberg, 1954*d*) a greater tendency to Group I lung cancer in males was observed in individuals with " dusty " occupations and trades, as well as in " clerical " and " professional " workers, when compared to individuals with " open air " and " house " work. In that paper it was underlined that the dust, *per se*, might be of importance, but it was also mentioned that other factors, in the line of life habits, more or less closely associated with different occupations, might be involved. Among the life habits tobacco smoking holds a prominent place.

In Table VIII a survey is given, in grams, of the smoking levels in the different occupations in the Group I tumours in males, arranged also according to domicile. The material is, admittedly, small and difficult to analyse. It seems, however, that at least there is no indication that the comparatively higher occurrence of Group I tumours in males in " dusty " trades and occupations is associated with an especially high smoking level. The figures may rather be interpreted as showing that " dusty " occupations may add another carcinogenic insult to the effect of the smoking. It cannot therefore theoretically be denied that besides the well known lung carcinogenic agents, such as nickel, asbestos, coal tar and uranium ore, some other " dusts " may be active and of importance in the development of some cases of lung cancer, even if the agent is so weak as not to be easily discovered. Exposure to metal dusts may especially be noted. It is worth noting, however, that among the 92 cases in " dusty " occupations there is not a single non-smoker.

" Clerical " and " professional " workers in the male Group I tumour material seem to be rather heavy smokers. Not one smoked less that 5 grams, and only 8 out of 53 patients smoked less than 10 grams a day. Non-smokers were not found.

The " open air " and " house " workers represent the largest occupational category, but they show the lowest number of Group I tumour cases—44 cases in all. It seems that also the tobacco smoking is on a lower general level than in the other occupational categories. This holds good not only for the relative number of smokers, but also for the amount smoked. Furthermore, it is interesting to note that

TABLE VIII.—*Smoking Levels in the Different Occupational Categories and in the Different Types of Domicile in Group I Tumour Patients. Males.*

| | Amount tobacco smoked in grams. | | | | | | | | | |
|---|---|---|---|---|---|---|---|---|---|---|
| | 0. | 1–4. | 5–9. | 10–14. | 15–19. | 20–24. | 25–29. | 30–49 | 50 +. | Total. |
| *Countryside* | | | | | | | | | | |
| " Open air " activities and " house " work | 3 | — | 3 | 3 | 1 | 1 | 2 | — | — | 13 (10) |
| " Clerical " and " professional " work | — | — | 1 | 3 | 2 | 1 | — | — | — | 7 |
| " Dusty " occupations | — | — | 3 | 4 | 1 | 2 | 1 | — | — | 11 |
| | 3 | — | 7 | 10 | 4 | 4 | 3 | — | — | |
| *Non-industrial towns* | | | | | | | | | | |
| " Open air " activities and " house " work | — | — | 2 | 7 | — | 1 | 1 | 1 | 1 | 13 |
| " Clerical " and " professional " work | — | — | 1 | 2 | 1 | 1 | 1 | 4 | — | 10 |
| " Dusty " occupations | — | — | 7 | 7 | 2 | 1 | 2 | 3 | — | 22 |
| | — | — | 10 | 16 | 3 | 3 | 4 | 8 | 1 | |
| *Industrial towns and centres* | | | | | | | | | | |
| " Open air " activities and " house " work | — | 1 | — | 3 | — | — | — | — | — | 4 |
| " Clerical " and " professional " work | — | — | — | 1 | 3 | 1 | 1 | 1 | — | 7 |
| " Dusty " occupations | — | 1 | 3 | 7 | 2 | 1 | — | 1 | — | 15 |
| | — | 2 | 3 | 11 | 5 | 2 | 1 | 2 | — | |
| *Larger towns (Oslo, Bergen, Trondheim)* | | | | | | | | | | |
| " Open air " activities and " house " work | — | 1 | 3 | 5 | 3 | 1 | 1 | — | — | 14 |
| " Clerical " and " professional " work | — | — | 6 | 4 | 9 | 2 | 2 | 4 | 2 | 29 |
| " Dusty " occupations | — | 1 | 7 | 23 | 3 | 5 | 2 | 2 | 1 | 44 |
| | — | 2 | 16 | 32 | 15 | 8 | 5 | 6 | 3 | |

all the 3 non-smokers in the total material of male Group I tumour patients belong to this category. The lung history of these 3 cases is as follows ;

(1) Farmer, 75 years old (Hordaland Fylke). No serious lung symptoms until two years before his death from a squamous cell carcinoma of the lung.

(2) Farmer, 67 years old (Hedmark Fylke). Since 1914 suffering from asthma with constant use of adrenaline. In 1951 a short story of increased lung symptoms, with the subsequent diagnosis of a squamous cell carcinoma.

(3) Fisherman, 67 years old (Akershus Fylke). During the winter of 1953 some dry coughing, in March pains and haemorrhage, in May diagnosis of a squamous cell carcinoma.

It may also be worth noting that all these patients belonged to age-groups above that with the maximum occurrence of Group I tumour cases.

Sailors and patients with unknown domicile have not been included in this part of the analysis.

In this connection it may be of interest to recall that in the study of the geographical distribution of the Norwegian lung cancer material, it was observed that Group I tumours were more heavily represented in the " smaller non-industrial towns " with " fresh air ", than in the " smaller industrial towns and centres " with more or less polluted air. This finding was initially unsuspected by the writer, on the background of the predominant assumption that general air pollution is of some importance for the development of lung cancer. The original paper comprised then 235 cases of lung cancer. The figures of the present material, augmented to 300 cases, confirm the previous observation.

The original " control " material did not give any information as to smoking habits in different types of domicile, although the finding of more moderate smoking in rural districts was expected. After the publication of the paper on geographical distribution, some other material with a bearing on the Norwegian smoking habits was placed at my disposal through the kindness and the generosity of " Fakta ", Oslo, an institute for marketing research. This institute had just completed a survey for one of our tobacco factories, and in the Appendix, Table III, some data are recorded, and in the text Fig. 1 presents the picture of the total smoking as well as the different types of smoking in areas corresponding to our " Rural districts ", "Smaller non-industrial towns ", " Smaller industrial towns and centres " and " Larger towns ". The figures presented are not comparable to the figures of our original " control " material, because of different criteria used. In our original " control " material, as well as in our lung cancer material, a smoker was defined as " a person who has smoked as much as 1 g. of tobacco (in any form) daily for at least one year ", whereas in the " Fakta " material, only present smokers are regarded as smokers. This will explain the very important differences as regards the percentages of smokers and non-smokers in the two series. The smoking types in the different geographical areas of the " Fakta " material are, however, comparable *inter se*, and Fig. 1 shows that the smoking habits in the smaller towns differ markedly. The percentage of smokers is practically the same, but the " Smaller non-industrial towns ", which mainly represent commercial and administrative centres, show a considerably higher number of pure and mixed cigarette smokers than the " Smaller industrial towns and centres ", even if the average number of grams smoked is the same (Appendix, Table III). The male Group I tumour patients show a heavier type of smoking in the former, as compared to the latter, as will be seen from Table VIII.

In Table IX the types of smoking are given for the male Group I and Group II tumour patients. There is a heavier representation of pure and mixed cigarette

TABLE IX.—*Different Smoking Types of Group I and Group II Tumour Patients (Males).*

| | Amount smoked in grams. | | | | | | | | | |
|---|---|---|---|---|---|---|---|---|---|---|
| Type of smoking. | 0. | 1–4. | 5–9. | 10–14. | 15–19. | 20–24. | 25–29. | 30–49. | 50 +. | Total. |
| **Group I.** | | | | | | | | | | |
| Non-smokers | 3 | — | — | — | — | — | — | — | — | 3 |
| Pure pipe smokers | — | 3 | 11 | 19 | 1 | 2 | — | — | — | 36 |
| Pipe and cigarettes | — | — | 12 | 18 | 11 | 4 | 5 | 3 | 4 | 57 |
| Pure cigarettes | — | 1 | 18 | 41 | 17 | 14 | 10 | 15 | 1 | 117 |
| **Group II.** | | | | | | | | | | |
| Non-smokers | 3 | — | — | — | — | — | — | — | — | 3 |
| Pure pipe smokers | — | — | 6 | 4 | 1 | 1 | 1 | — | — | 13 |
| Pipe and cigarettes | — | — | 3 | 2 | 1 | — | 1 | — | — | 7 |
| Pure cigarettes | — | 5 | 5 | 6 | 3 | — | 2 | — | 1 | 22 |

smokers in the Group I series, and more pipe smokers in the Group II. The Group I series, as regards cigarette smoking, shows considerably higher figures than the Norwegian " control " material presented by Kreyberg (1954). A detailed comparison with the " Fakta " material is useless because of the different criteria used for

FIG. 1.—The smoking habits of males of different types of domicile, " Fakta " material. The columns show percentage smokers in total, as well as separately for pure pipe smokers (P), mixed pipe-cigarette smokers (M) and pure cigarette smokers (C), in rural districts (R), smaller non-industrial towns (RT), smaller industrial towns and centres (F.T) and larger towns (L.T). P ; M ; C .

non-smokers. If one examines the ratio Group I : Group II tumours in pure pipe smokers, pipe and cigarette smokers and pure cigarette smokers, one finds figures as shown in Table X.

TABLE X.—*Ratio Group I : Group II Tumours Males. Different Types of Smoking.*

| Amount smoked. | None. | Pipe only. | Pipe and cigarettes. | Cigarettes only. |
|---|---|---|---|---|
| Under 10 g. | — | 2·3 : 1 | 4·0 : 1 | 1·9 : 1 |
| 10–19 ,, | — | 4·0 : 1 | 9·7 : 1 | 6·4 : 1 |
| 20 + ,, | — | 1·0 : 1 | 16·0 : 1 | 13·3 : 1 |
| Total | 1 : 1 | 2·8 : 1 | 6·0 : 1 | |

The absolute figures are small and the picture is not completely clear, therefore no precise conclusions can be drawn, but it may be worth calling attention to the seemingly lacking increase in the ratio of the pure pipe smokers to the increasing amount of tobacco smoked, in contrast to the definite and systematic increase in the ratio when cigarette smoking is involved. This may point in the direction that pure pipe smoking actually is connected with a more moderate risk, as regards lung cancer development, than is cigarette smoking. Furthermore, on the average the pure pipe smokers do not seem to use as great amounts of tobacco as do the cigarette smokers, and a quantitative factor may possibly also be of importance in this respect.

One feature from the tables reproduced is very striking, namely, the different occurrence of some types of lung cancer in the two sexes, even among the non-smokers. As there are many more non-smokers among the females, and as many female smokers are very moderate smokers, the absolute figures cannot be compared. But, if the ratio Group I tumours to Group II tumours is again used as an analytical method on the male and female non-smokers, one finds the figures 1 : 1 in males (3 : 3 cases), and 1 : 9 in females (3 : 27 cases) (Table XI).

If the figures for smokers at the level of less than 5 grams, where no carcinogenic association has been observed in our material, are also included, essentially the same picture is found; a ratio for males 1 : 1·1 (7 : 8 cases), and for females 1 : 7·5 (4 : 30 cases). The basic figures are found in Table VI.

TABLE XI.—*Sex Differences. Ratio Group I : Group II Tumours.*

| | | |
|---|---|---|
| Male non-smokers | 1 : 1 | (3 : 3) |
| „ smokers | 5 : 1 | (210 : 42) |
| Female non-smokers | 1 : 9 | (3 : 27) |
| „ smokers | 1 : 5 | (2 : 10) |

With due allowance for the smallness of the numbers, this shows that even in non-smokers the males dominate the Group I tumours markedly. Wynder (1954), in his Table VI presents—from his own material and from the literature—a number of figures showing the occurrence of types of lung cancers (very much like our own grouping) in non-smoking males, and from that material one arrives at a ratio 2·4 : 1, a very low ratio, even if not as low as the Norwegian one quoted above.

The ratio "Epidermoid" carcinomas (including "oat-cell" carcinomas); "Adenocarcinomas" (including bronchiolar cell carcinomas) from Wynder's collected material differs from my present material, the explanation being that adenomas and salivary gland tumours are also included in my Group II tumours. If these are excluded, my material shows a ratio 1·8 : 1, very close to Wynder's.

In addition, the ratios are given from four Norwegian post-mortem series, partly from the periods when the new carcinogenic situation was not yet manifested (Table XII). In this table all cases are included, smokers as well as non-smokers. The different figures quoted seem altogether to strengthen the above conclusion that, in Norway at present, the ratio Group I : Group II tumours in male non-smokers is very nearly 1 : 1.

TABLE XII. *Ratio Group I: Group II Tumours in Males in Four Norwegian Post-mortem Series.*

| Material. | Number of cases. | | Ratio. |
|---|---|---|---|
| Christiansen I R. H. (1925–44) | 25 | 15 | 1·7 : 1 |
| Jacobsen Ullevål (1937–46) | 46 | 24 | 1·9 : 1 |
| Christiansen II R. H. (1945–52/1) | 40 | 14 | 2·9 : 1 |
| Kreyberg (1948–55) | 213 | 45 | 4·7 : 1 |

These figures may be supposed to be in contrast to the statement by Doll (1953); "That the incidence of lung cancer in non-smokers may be the same in men and in women and in residents in areas of different density of population," a statement with which Wynder agrees. Our Norwegian figures may initially seem more in agreement with the standpoint of Fischer (1954/55), who comments upon Doll's conclusion as follows ; " I have the opinion, from the critically digested available information, that such a conclusion cannot be drawn."

Possibly the contrasting statements may be reconciled. It should be emphasised that the very marked sex difference in the Norwegian material just mentioned concerns the Group I tumours only. If the figures from Table XI are used to compare all types of lung cancer, seen as an entity—and that is the background of Doll's analysis—one finds a total of 6 " lung cancers " (3 + 3) in the non-smoking males and 30 cases (3 + 27) in the non-smoking females. As the non-smoking female part of the population however is very much greater than the corresponding male part, a factor of 5 is very likely according to our previously quoted figures, one arrives at an approximately equal sex occurrence of " lung cancer " total among non-smokers. As Group I tumours in the Norwegian material are rather uncommon among females at all, as well as among non-smoking males, the peculiar sex difference in the occurrence in the Group I tumours is easily obscured by the great number of Group II tumours if special attention is not paid to the histological typing, followed by a separate treatment of the two groups. Again, it seems that the value of this analytical procedure, when fully used, has been demonstrated.

The enigma of the preponderance of male victims of Group I tumours in non-smokers is further emphasized in the Norwegian material by the fact that all cases (admittedly only 3) were among the " open air " workers (2 farmers and 1 fisherman), with not a single case of Group I tumours belonging to the categories " dusty ", " clerical " or " professional " workers.

This means that the possibility of a special sex disposition to the development of Group I tumours in males cannot be denied. Whether this disposition is based upon architectural differences in the gross anatomy of the lungs, upon different biological responses of the epithelium, or upon hormonal factors is completely unknown, and the question is at present unassailable by clinical and experimental methods. Gross anatomical studies however, are possible.

Such a sex factor may even be operative in cases where a most probable special causative factor has been found, as seems to be the case regarding tobacco smoking. From the literature it seems that the decided increase in male lung cancers, and that means Group I tumours, started at a time when cigarette smoking was not very prevalent. Considering a latency period, or a period of development, of some 20 to 40 years, the changed carcinogenic situation should, accordingly, have commenced at the end of the last, or at the very beginning of this century for a series of countries. If the female response to tobacco smoking is the same as that of the male it would accordingly be reasonable to-day to expect the finding of a definite increase in female Group I tumours in the countries leading in this respect. A critical study of the literature does not reveal with certainty that such an increase has actually taken place.

### SUMMARY AND CONCLUSIONS

In a series of previous papers it has been shown that an increase in lung cancer in males has taken place in Norway in this century, closely following the pattern of development in a number of other countries, although, it seems, with the delay of a decade or two.

In the beginning of this century a slow and parallel rise in the number of cases of lung cancer registered in males and females was found, but gradually, and from the middle of the nineteen forties especially, the increase in male cases has greatly exceeded the increase in female cases.

A study of the histological types of lung cancer has revealed that from the period of the more pronounced increase in male cases, the relative frequency of the histological types has also changed, with a steadily increasing number of Group I tumour cases (squamous cell, large cell and small cell carcinomas) in males. No such change has been found in females. It has previously been concluded that nearly the whole increase in female cases, and part of the increase in male cases, which has been registered, is caused by better diagnostic facilities, whereas the specific increase in Group I tumour cases in males is caused by a real increase in the development of lung tumours. This means that a new carcinogenic situation has been established, which in Norway manifested itself definitely in the middle of the nineteen forties. As the Group I tumour cases in males have a period of development ranging between twenty and forty years or more, the new carcinogenic situation should, therefore, have commenced early in this century.

This new carcinogenic situation, up to the present moment, has been manifested through male victims only. From theoretical considerations, as well as from our special studies, it is regarded as unlikely that this new carcinogenic situation is based upon a general air pollution, even if its effects are mainly observed in urban areas. The main active principle(s) should be sought in the closer environments of the males, most probably in connection with the male's working conditions and/or his life habits.

Whereas it is well known that certain occupations, connected with radio-active material, nickel, asbestos and coal tar, are associated with an increased lung cancer development, the Norwegian material does not show any special trade or occupation with such a marked risk that the increased occurrence of Group I tumour cases in males can be explained solely by occupational hazards.

Admittedly males in " dusty " occupations generally have an increased lung cancer frequency as compared to " open air " workers. But nearly as great a risk is also noted among " clerical " and " professional " workers.

A study of the tobacco smoking habits of the lung cancer cases has now been conducted in such a manner that the habits of the general population, the habits of the Group I tumour cases, as well as those of the Group II tumour cases (adenocarcinomas, bronchiolar cell carcinomas, adenomas benign and malignant, and salivary gland tumours of the lungs), have been analysed separately for each group and for each sex, and the figures compared.

The material has consisted of 213 Group I and 45 Group II tumour cases in males, and 5 Group I and 37 Group II tumour cases in females. The main conclusions of this study are as follows ;

(1) As no differences have been found, either in males or in females, between the smoking habits of the Group II tumour patients and the smoking habits of the population in general, it is concluded that tobacco smoking has no relationship to the occurrence of Group II lung tumours. Such tumours represent, in Norway to-day, nearly 90 per cent of all female lung tumour cases, but less than 20 per cent of all male cases.

(2) As a considerably lower number of non-smokers are found among the male Group I lung tumour patients than among the male Group II tumour patients, as well as among the corresponding male " control " material, and as a consistently and steadily increasing ratio of Group I : Group II cases is observed with the increasing amounts of tobacco smoked, it is concluded that tobacco smoking is closely related to the development of a considerable proportion of the Group I tumour cases in males. The very limited female material does not present any contradictory facts invalidating this conclusion.

(3) As a certain number of Group I tumour cases occur in male and in female non-smokers, it is concluded that not all Group I tumour cases are caused by, or are influenced in, their development by tobacco smoking.

(4) From an analysis of the ratio Group I : Group II tumours in males, it has been calculated that, in Norway at present, four out of five cases of Group I lung tumours in males are related to tobacco smoking, and that one out of five cases arises from causes unrelated to tobacco smoking.

(5) As males in " dusty " work show the relatively greatest number of Group I lung tumours in spite of a more moderate tobacco consumption than shown by the Group I lung tumour patients among " clerical " and "professional " workers, it may tentatively be suggested that industrial dusts and fumes add an aggravating factor to the injury caused by tobacco smoking, as regards the development of lung cancer.

(6) A previously recorded finding of relatively more Group I tumour cases in males in smaller non-industrial towns (administrative and commercial centres) with " fresh air ", than in smaller industrial towns and centres, with more, or less, polluted air, may be explained by the finding of more pure and mixed cigarette smokers in the former as compared to the latter type of community.

(7) The relationship between pipe smoking and lung cancer development is much less marked than the relationship between cigarette smoking and lung cancer.

(8) The ratio Group I : Group II tumours in non-smokers is nine times greater in the males than in the females. This very great difference, *per se*, as well as the fact that the only three male Group I tumour non-smokers, registered in the present material, occurred among the " open air " workers, makes it difficult to ascribe the whole sex difference in the occurrence of Group I tumours to external factors alone. A biological sex difference, architectural and/or biochemical, influencing the response of the lungs to some, or all, of the principles causing lung cancer, cannot be excluded.

After this discussion, and after the conclusions presented, it may be useful to underline the following points.

Firstly, the conclusions are based upon a special histological subdivision of the " lung cancer " material, according to certain definite criteria. If this grouping and these criteria used are not followed the same results cannot be obtained. An off-hand denial of the possibility of making a sufficiently precise histological grouping is dismissed by the results. Not every case can be grouped with certainty, but most cases can.

Secondly, the results of the present study are so systematic and so clear that it is unreasonable not to consider the findings as expressions of true conditions. An artefact with such a degree of consistency is most improbable.

Thirdly, the figures quoted represents trends and relationships only. They do not express fixed mathematical correlations generally applicable. The figures are approximate and applicable to the conditions in Norway during the period of investigation, and cannot be applied to other countries and to other conditions without further qualifications. The trends of the general conclusions I do, however, regard as generally valid.

The present investigation has been aided by a generous grant from " Tobaksfabrikermes Landsforening av 1901 "

My sincere thanks are also due to the " Fakta " Marketing Research Institute, Oslo, for valuable statistical material, as regards smoking habits in Norway to-day.

I am likewise greatly indebted to Dr. R. Doll, London, and Dr. R. Korteweg, Amsterdam, for valuable discussions during the preparation of the manuscript.

### REFERENCES

BRESLOW, L., HOAGLIN, L., RASMUSSEN, G. AND ABRAMS, H.—(1954) *Amer. J. Publ. Hlth.*, **44**, 171.

DOLL, R.—(1953) *Brit. J. Cancer*, **8**, 303.—(1955) ' Advances in Cancer Research.' New York (Academic Press Inc.).

*Idem* AND HILL, A. B.—(1952) *Brit. med. J.*, ii, 1271.

FISCHER, W.—(1954/55) *Wiss. Z. Fried. Schiller Universitaet Jena*, **4**, 21.

HAMMOND, E. C. AND HORN, D.—(1955) Stenciled report from the Annual Meeting of the American Medical Association in Atlantic City, June 6.

KREYBERG, H. J. A.—(1954) *Brit. J. Cancer*, **8**, 13.

KREYBERG, L.—(1952) *Ibid.*, **6**, 112.—(1954*a*) *Ibid.*, **8**, 199.—(1954*b*) *Ibid.*, **8**, 209.—(1954*c*) *Ibid.*, **8**, 599.—(1954*d*) *Ibid.*, **8**, 605.

LEVIN, M. L.—(1954) *N.Y. St. J. Med.*, **54**, 769.

LICKINT, F.—(1953) ' Ätiologie und Prophylaxe des Lungenkrebses.' Dresden und Leipzig (Steinkopff).

RANDIG, K.—(1954) *Öff. GesundhDienst.*, **16**, 305.

WYNDER, E. L.—(1954) *Penn. med. J.*, **57**, 1073.

*Idem* AND GRAHAM, E. A.—(1950) *J. Amer. med. Ass.*, **143**, 329.

APPENDIX TABLE I.—*Smoking Habits of the " General Population ", Based upon an Aggregation of Groups* 1, 2, 4, 5, 6 (*M*) *and Groups* 1, 2, 3, 4, (*F*), *Excluding Persons* 85 *Years and Older, from Kreyberg* (1954).

*Males.*

| | Non-smokers. | | Smoking in grams. 1–14. | | 15–24. | | 25 or more. | | Total. | |
|---|---|---|---|---|---|---|---|---|---|---|
| Age. | No. | Per cent. | No. | Per cent. | No. | Per cent. | No. | Per cent. | No. | Per cent. |
| 35 | 268 | 20·92 | 716 | 55·89 | 240 | 18·74 | 57 | 4·45 | 1281 | 100·00 |
| 35–44 | 176 | 14·03 | 680 | 54·23 | 322 | 25·68 | 76 | 6·06 | 1254 | 100·00 |
| 45–54 | 102 | 10·66 | 520 | 54·34 | 263 | 27·48 | 72 | 7·52 | 957 | 100·00 |
| 55–64 | 58 | 13·33 | 272 | 62·53 | 77 | 17·70 | 28 | 6·44 | 435 | 100·00 |
| 65–84 | 40 | 17·32 | 153 | 66·23 | 23 | 9·96 | 15 | 6·49 | 231 | 100·00 |
| Total | 644 | | 2341 | | 925 | | 248 | | 4158 | |

*Females.*

| | Non-smokers. | | Smoking in grams. 1–14. | | 15–24. | | 25 or more. | | Total. | |
|---|---|---|---|---|---|---|---|---|---|---|
| Age. | No. | Per cent. | No. | Per cent. | No. | Per cent. | No. | Per cent. | No. | Per cent. |
| 35 | 237 | 58·52 | 151 | 37·28 | 17 | 4·20 | 0 | 0·00 | 405 | 100·00 |
| 35–44 | 124 | 60·79 | 66 | 32·35 | 13 | 6·37 | 1 | 0·49 | 204 | 100·00 |
| 45–54 | 131 | 69·31 | 50 | 26·46 | 6 | 3·17 | 2 | 1·06 | 189 | 100·00 |
| 55–64 | 111 | 87·40 | 13 | 10·24 | 3 | 2·36 | 0 | 0·00 | 127 | 100·00 |
| 65–84 | 54 | 90·00 | 6 | 10·00 | 0 | 0·00 | 0 | 0·00 | 60 | 100·00 |
| Total | 657 | | 286 | | 39 | | 3 | | 985 | |

APPENDIX TABLE II.—*Smoking Levels of Male Group I and Group II Tumour Patients (Age-Group 45–54 and 55–64 only) and "Controls" Matched to Age-Groups.*

| | Non-smokers. | Smoking in grams. 1–14. | 15–24. | 25 +. | Total cases. |
|---|---|---|---|---|---|
| Group I | 0 | 91 | 40 | 30 | 161 |
| " Control " (general population) | 19·5 | 94·9 | 35·4 | 11·2 | 161 |
| Group II | 1 | 15 | 2 | 4 | 22 |
| " Control " (general population) | 2·6 | 12·9 | 4·9 | 1·6 | 22 |

APPENDIX TABLE III.—*Norwegian Smoking Habits.*

(From a market analysis by " Fakta ")

| | Rural districts. | Smaller non-industrial towns. | Smaller industrial towns and centres. | Larger towns. |
|---|---|---|---|---|
| *Males.* | | | | |
| Number examined | 643 | 264 | 279 | 783 |
| Per cent smokers (total) | 62 | 77 | 76 | 77 |
| ,, pipe smokers | 24 | 13 | 24 | 16 |
| ,, mixed cigarette smokers | 19 | 26 | 31 | 27 |
| ,, pure cigarette smokers | 19 | 38 | 21 | 34 |
| Average number grams per day per smoker | 8·6 | 10·7 | 10·6 | 10·3 |
| Per cent smokers each age group : | | | | |
| 30 years and younger | 74 | 91 | 88 | 79 |
| 30–44 years | 72 | 90 | 79 | 81 |
| 45–59 years | 51 | 62 | 69 | 80 |
| 60 years and more | 49 | 70 | 65 | 63 |
| *Females.* | | | | |
| Number examined | 723 | 262 | 278 | 833 |
| Per cent smokers (all cigarette) | 10 | 31 | 25 | 35 |
| Per cent smokers each age group : | | | | |
| 30 years and younger | 28 | 45 | 26 | 35 |
| 30–44 years | 13 | 37 | 39 | 45 |
| 45–59 years | 2 | 24 | 14 | 29 |
| 60 years and more | 2 | 7 | — | 12 |